# Arginine Methyltransferases as Regulators of RNA-Binding Protein Activities in Pathogenic Kinetoplastids

**DOI:** 10.3389/fmolb.2021.692668

**Published:** 2021-06-11

**Authors:** Gustavo D. Campagnaro, Edward Nay, Michael J. Plevin, Angela K. Cruz, Pegine B. Walrad

**Affiliations:** ^1^ Department of Cell and Molecular Biology, Ribeirão Preto Medical School, University of São Paulo, Ribeirão Preto, Brazil; ^2^ York Biomedical Research Institute, Department of Biology, University of York, York, United Kingdom

**Keywords:** arginine methylation, PRMT, RNA-binding protein, *Trypanosoma*, *Leishmania*, gene expression, Kinetoplastid, post-translational modification

## Abstract

A large number of eukaryotic proteins are processed by single or combinatorial post-translational covalent modifications that may alter their activity, interactions and fate. The set of modifications of each protein may be considered a “regulatory code”. Among the PTMs, arginine methylation, catalyzed by protein arginine methyltransferases (PRMTs), can affect how a protein interacts with other macromolecules such as nucleic acids or other proteins. In fact, many RNA-binding (RBPs) proteins are targets of PRMTs. The methylation status of RBPs may affect the expression of their bound RNAs and impact a diverse range of physiological and pathological cellular processes. Unlike most eukaryotes, Kinetoplastids have overwhelmingly intronless genes that are arranged within polycistronic units from which mature mRNAs are generated by *trans*-splicing. Gene expression in these organisms is thus highly dependent on post-transcriptional control, and therefore on the action of RBPs. These genetic features make trypanosomatids excellent models for the study of post-transcriptional regulation of gene expression. The roles of PRMTs in controlling the activity of RBPs in pathogenic kinetoplastids have now been studied for close to 2 decades with important advances achieved in recent years. These include the finding that about 10% of the *Trypanosoma brucei* proteome carries arginine methylation and that arginine methylation controls *Leishmania*:host interaction. Herein, we review how trypanosomatid PRMTs regulate the activity of RBPs, including by modulating interactions with RNA and/or protein complex formation, and discuss how this impacts cellular and biological processes. We further highlight unique structural features of trypanosomatid PRMTs and how it contributes to their singular functionality.

## Introduction

**FIGURE 1:**
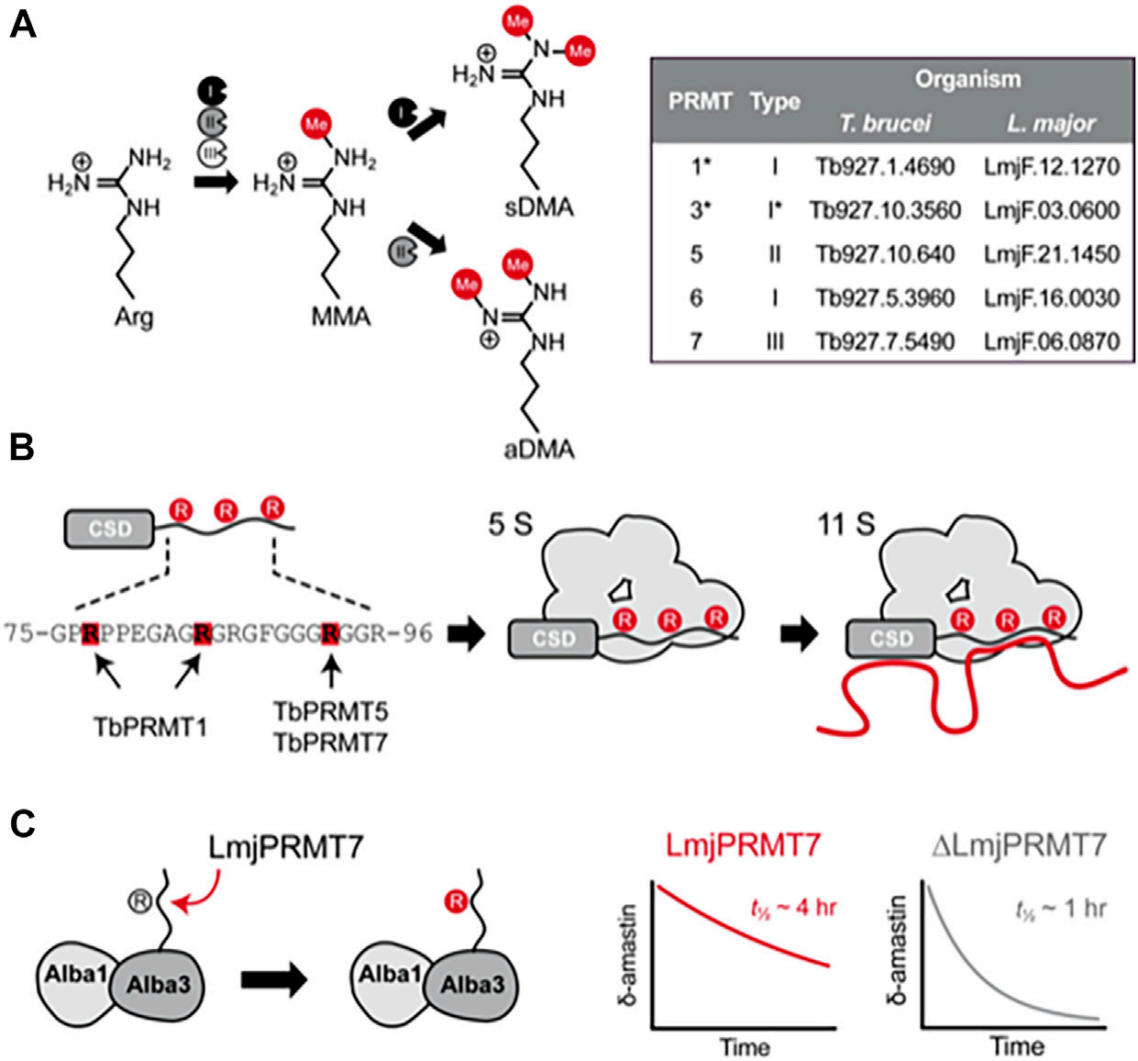
Molecular effects of arginine methylation in *T. brucei* and *L. major*. **(A)**, Protein arginine methyltranferases (PRMTs) from types I, II and III are able to generate monomethylarginine (MMA) by transfering the methyl group from S-adenosylmethionine to the terminal nitrogen atom of arginine residues. While type III PRMTs only produce monomethylated products, type I PRMTs catalyze a second round of methylation at the same atom, generating asymmetrically dimethylated arginine (aDMA), whereas type II PRMTs add another methyl group to the adjacent terminal nitrogen, forming symmetric dimethylarginine (sDMA). The inset table contains the gene IDs of PRMT genes found in the genome of *T. brucei* and *L. major*. **(B)**, Schematic representation of how methylation affects the capability of RBP16 to form macromolecular complexes containing proteins (gray) and RNA (red line) in *T. brucei*. The RBP16 intrinsically disordered RGG domain is methylated by *Tb*PRMT1 on Arg78 and Arg85, whereas Arg93 is (potentially) methylated by either or both *Tb*PRMT5 and *Tb*PRMT7 **(left)**. In its methylated state, RBP16 can associate with other proteins (5S complex) or with proteins and RNA (11S complex). A non-methylatable version of RBP16 is still able to associate with RNA but loses the capability to form multiprotein complexes. Non-methylated arginines are represented by gray circles and methylated arginines by red circles. **(C)**, Representation of the methylation mediated by *L. major* PRMT7 on Alba3. Alba3 interacts with Alba1 and δ-amastin transcripts. Methylated Alba3 has a stronger association with δ-amastin transcripts and protects the RNA from degradation. The ability of Alba3 to bind δ*-amastin* is reduced upon *Lmj*PRMT7-knockout, which reduces the half-life of the transcripts from approximately 4 h to around 1 h. *PRMT3 is currently known as PRMT1^PRO^ in *T. brucei*; despite its similarity to mammalian PRMT3, *Tb*PRMT3 misses key residues for PRMT activity, and is rather a prozyme for the catalytic *Tb*PRMT1, which was thus renamed to *Tb*PRMT1^ENZ^.

A large number of eukaryotic proteins are processed by single or combinatorial post-translational modifications (PTMs) that may alter protein function, conformation, localization, and/or their interaction with other macromolecules (Tak et al., 2019). The methylation of arginine residues is mediated by Protein Arginine Methyltransferases (PRMTs), of which three types exist: I, II and III. All three types generate ω-monomethylarginine (MMA) by transferring the methyl group from S-adenosylmethionine (SAM) to a ω-nitrogen atom. Type I PRMTs can add a further methyl to the same nitrogen to form asymmetric ω-dimethylarginine (aDMA) while type II PRMTs modify the other ω-nitrogen to generate symmetric ω-dimethylarginine (sDMA) (Bedford, 2007) (Figure 1A). PRMTs show a preference for arginine-glycine (RGG) rich motifs, which are commonly enriched in intrinsically unstructured regions of proteins and implicated in RNA binding and biomolecular liquid-liquid phase separation (Chong et al., 2018).

Kinetoplastida are the only parasitic protozoa to harbor genes encoding for PRMTs of types I, II and III (Fisk and Read, 2011) (Figure 1A). This group of early branching eukaryotes includes the causative agents of important human diseases: Sleeping Sickness (*Trypanosoma brucei*), Chagas disease (*Trypanosoma cruzi*) and the leishmaniases (*Leishmania* spp.). During their life cycles, trypanosomatids alternate between several morphologically and metabolically different stages, which requires fine-tuned regulation of gene expression.

As part of the class Kinetoplastea, trypanosomatids display some particular features, such as the arrangement of genes in long polycistronic transcription units (PTUs) and *trans*-splicing of all mRNAs (Adl et al., 2019). Given the lack of individual promoters and terminators, virtually all genes in Kinetoplastids are constitutively transcribed as part of PTUs (Clayton, 2016; Damasceno et al., 2020), which makes these parasites good models for the study of mechanisms involved in post-transcriptional gene regulation.

A multitude of studies have shown that the levels of RNA-binding proteins (RBPs) fluctuate throughout the life cycle of trypanosomatids, some being stage-specific, even dictating the transition from one biological form to another (Kolev et al., 2014; de Pablos et al., 2019). Less, however, is understood about the mechanisms regulating the activity of RBPs. In this sense, arginine methylation has been gaining attention as a regulatory mechanism of nucleic acid-binding protein activities in trypanosomatids (Lott et al., 2013; Ferreira et al., 2020). In fact, knowledge of trypanosomatid PRMTs has grown substantially in the recent years, with the disclosure of protein structures, and the determination of the molecular effects of arginine methylation, particularly on RBPs. The differences observed in the biochemical, biophysical and structural properties of trypanosomatid PRMTs in comparison to their mammalian counterparts suggest that PRMTs may be good targets for drug development.

Here, we provide an up-to-date review of the activities of PRMTs in pathogenic trypanosomatids, as well as discuss the effect of arginine methylation in cellular and molecular processes, particularly on the function of RBPs. Moreover, we discuss the structural features of trypanosomatid PRMTs and how these might enable revised design and repurposing of current drugs to combat these parasites.

## Protein Arginine Methyltransferases in *Trypanosoma*

The first evidence for arginine methylation in *T. brucei* dates from 1991 (Yarlett et al., 1991), that was directly confirmed a decade later (Pelletier et al., 2001). Recently, the use of high-throughput techniques revealed that close to 10% of the *T. brucei* proteome harbors methylated arginines (Lott et al., 2013), the product of the cooperative action of five PRMTs found in its genome (Lott et al., 2014). Nomenclature of *Trypanosoma* proteins corresponds to the human PRMTs.

### *Trypanosoma brucei* Protein Arginine Methyltransferase 1, the First Discovered in Kinetoplastids

The first *T. brucei* protein identified to harbor methylated arginine residues was RBP16, a protein involved in mitochondrial RNA processing (Hayman and Read, 1999; Pelletier and Read, 2003). Three arginine residues, Arg-78, Arg-85 and Arg-93, are part of the RGG domain of RBP16 and methylation influences RBP16-RNA interactions (Pelletier et al., 2000; Miller and Read, 2003). Of these, only Arg-93 is constitutively methylated, while methylation of Arg-78 or Arg-85 appears to be mutually exclusive.

*In vitro* methylation assays using recombinant RBP16 and *T. brucei* procyclic whole cell protein extracts in the presence of classic substrates of type I and type II PRMTs indicated RBP16 is methylated by a trypanosome type I PRMT (Pelletier et al., 2001). The search for the type I PRMT that methylates RBP16 led to the identification of a protein 51% identical to the human PRMT1, thus named *Tb*PRMT1, whose knockdown (KD) abolished RBP16^Arg78^ and RBP16^Arg85^ methylation (Pelletier et al., 2005; Goulah et al., 2006). Arg-93 remains methylated, likely due to the action of another PRMT. Curiously, *Tb*PRMT1 is mostly present in the cytoplasm, suggesting RBP16 might be methylated before import into the mitochondrion (Fisk et al., 2010).

In *T. brucei*, RBP16 forms complexes of various sizes, but most notably 5S and 11S complexes; the latter likely represents the proteinaceous 5S complex bound to RNAs. Curiously, cells depleted for *Tb*PRMT1 or expressing the R78K, R85K and R93K triple mutant RBP16 formed a 5S complex composed of RBP16 bound only to mitochondrial guide RNAs (gRNAs), implicit in RNA editing, but not mRNAs (Figure 1B). Accordingly, non-methylatable RBP16 has an increased affinity for gRNAs, yet displays lower affinity for mitochondrial mRNAs (Goulah and Read, 2007). It is, however, unknown which proteins interact with RBP16 and whether mitochondrial mRNAs bind directly to RBP16 in the complex. Nonetheless, expression of non-methylatable RBP16 has been associated with destabilization of *NADH dehydrogenase subunit 4* mRNA, whose quantity is also lower in *Tb*PRMT1-KD cells, though *Tb*PRMT1-knockdown also impacts levels of other mRNAs (Goulah et al., 2006). The effects of arginine methylation by *Tb*PRMT1 are not limited to the mitochondrion. DRBD18 is a cytoplasmic RBP whose methylation state leads to different protein complex formation and alters mRNA expression. RNAs stabilized by methylated DRBD18 are less stable in the absence of *Tb*PRMT1 or when non-methylatable DRBD18 is expressed; the opposite is true for RNAs destabilized by DRBD18 (Lott et al., 2015). In fact, *Tb*PRMT1 knockout has a broad, complex effect on mRNP associations, which impacts cell metabolism, particularly energy production pathways, as well as stress granule formation, and results in reduced *in vitro T. brucei* growth and virulence in mice (Kafková et al., 2018).

### *Trypanosoma brucei* Protein Arginine Methyltransferase 1 Activity Is Dependent on *Tb*PRMT3

Like *Tb*PRMT1, *Tb*PRMT3 was also identified as a potential type I PRMT and knockdown of either *Tb*PRMT1 or *Tb*PRMT3 reduced aDMA levels in the cells. Interestingly, the reduction in the protein level of either was accompanied by a reduction of the other, suggesting an interdependent stability between *Tb*PRMT1 and *Tb*PRMT3 (Pelletier et al., 2005; Lott et al., 2014).

**FIGURE 2:**
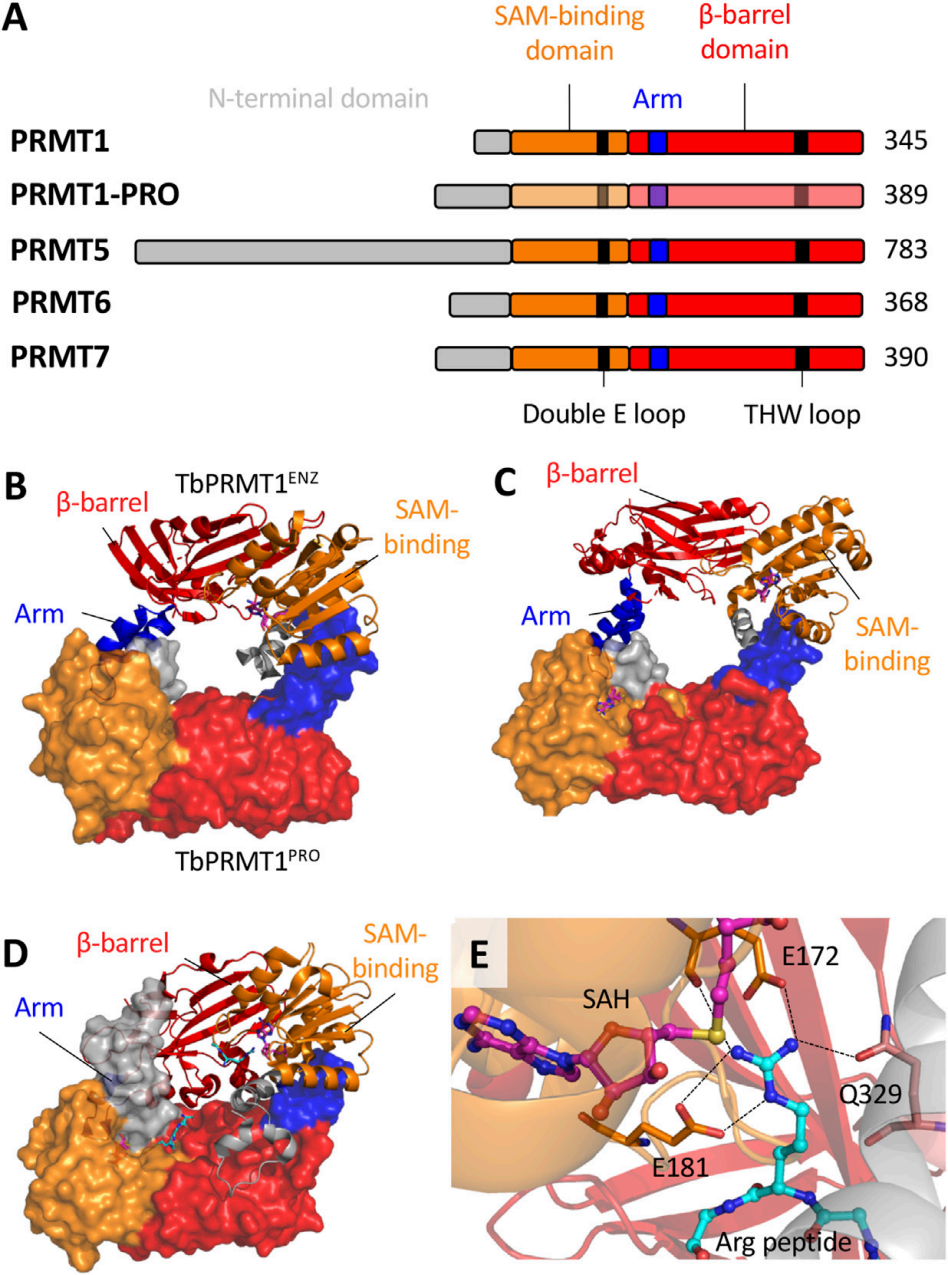
The structural biology of *T. brucei* PRMTs. **(A)**, Each of the five PRMT homologs in *T. brucei* contain the four canonical domains indicated. The SAM-binding domain contains the residues that interact with a SAM molecule and the target arginine substrate. The β-barrel domain contains residues that interact with the arginine substrate. The arm (dimerization arm) within the β-barrel domain interacts with another subunit via contacts to the SAM-binding domain. *Tb*PRMT N-termini have significant variability with elusive functional roles. Key conserved double E loop and THW loop are also indicated. B-D, The core dimeric interfaces of **(B)** *Tb*PRMT1^ENZ^-*Tb*PRMT1^PRO^ (PDB: 6DNZ) **(C)** *Tb*PRMT6 (PDB: 4LWP) and **(D)** *Tb*PRMT7 (PDB: 4M38). The surface structure represents the second subunit (or *Tb*PRMT1^PRO^ indicated in B. The SAH and Arg peptides are indicated by pink-blue-red and cyan-blue-red sticks respectively. **(E)** The active site of *Tb*PRMT7 (PDB: 4M38) as a representative. The SAH molecule and arginine substrate are indicated. The double E loop (E172 and E181) and THW loop (Q329) residues interact with the arginine substrate side chain. Dashed lines indicate hydrogen bonds. The combination of hydrogen bonds and electrostatic interactions from E172 and E181 with the arginine guanidino group result in strong salt bridges. Q329 forms a hydrogen bond to the guanidino group via its side chain amide oxygen.

However, *Tb*PRMT3 is inactive *in vitro* (Kafková et al., 2017), and its primary sequence lacks conserved residues in THW and double E loops, which are typically well conserved and responsible for substrate binding and positioning, respectively (Tewary et al., 2019). Structural data showed that although *Tb*PRMT3 retains the four canonical PRMT domains (N-terminus, SAM-binding Rossman fold domain, dimerization arm and β-barrel domain; Figure 2A), it lacks a crucial 3_10_ α-helix in the Rossman fold, which alters the dimerization interface and precludes SAM binding (Kafková et al., 2017; Hashimoto et al., 2020). Importantly, *T. cruzi* PRMT3 also lacks conserved THW and double E loops.

Functional studies have shown that *Tb*PRMT3 is essential for *Tb*PRMT1 stability and activity, establishing it as a “prozyme” or “pseudoenzyme” that supports the catalytically active *Tb*PRMT1. *Tb*PRMT1 and *Tb*PRMT3 have, thus, been renamed as *Tb*PRMT1^ENZ^ and *Tb*PRMT1^PRO^, respectively (Kafková et al., 2017; Hashimoto et al., 2020) (Figure 1A). Interestingly, when amino acids 41–52 were removed from *Tb*PRMT1^PRO^, methyltransferase activity was lost as the complex could no longer bind substrates. Although enzymatically inactive, *Tb*PRMT1^PRO^ contributes to substrate recognition (Hashimoto et al., 2020).

Unlike mammalian counterparts, *Tb*PRMT1^ENZ^ and *Tb*PRMT1^PRO^ interact via a hydrophobic interface to form a ring-like heterodimeric structure (Figure 2B), similar to homodimeric PRMTs. Two *Tb*PRMT1^ENZ^‒*Tb*PRMT1^PRO^ heterodimers together form the functional heterotetramer (Hashimoto et al., 2020). Curiously, *Tb*PRMT1^PRO^ can homodimerize in solution, albeit minimally (Kafková et al., 2017), whereas *Tb*PRMT1^ENZ^ is challenged to form homodimers (Hashimoto et al., 2020), supporting structural dependence on *Tb*PRMT1^PRO^.

It is possible that *Tb*PRMT1^PRO^ (alone or as a homodimer) performs moonlighting functions in the cell, as *Tb*PRMT1^PRO^ transcripts were slightly increased upon DNA damage induction, while *Tb*PRMT1^ENZ^ transcripts were reduced. *Tb*PRMT1^PRO^ has also been shown to bind mRNA both *in vitro* and *in vivo* independent of tetramer formation (Lueong et al., 2016; Stortz et al., 2017; Kafková et al., 2018). The biological relevance of proposed moonlighting properties remains unclear.

### *Trypanosoma brucei* Protein Arginine Methyltransferase 5

*Tb*PRMT5 is the only type II PRMT expressed by the parasite, yet the least studied *T. brucei* PRMT and the only one whose structure remains unsolved. *Tb*PRMT5 is by far the largest *T. brucei* PRMT due to a very long N-terminal region, the biological relevance of which is unknown (Figure 2A). *In vitro*, recombinant *Tb*PRMT5 displays a broad substrate specificity that includes RBP16 (Pasternack et al., 2007). Whether *Tb*PRMT5 methylates RBP16 *in vivo* is unknown, but if so, *Tb*PRMT5 may be responsible for constitutive methylation of RBP16^Arg93^. Moreover, the fact that recombinant *Tb*PRMT5 is active suggests that, unlike mammalian PRMT5, it does not require a co-factor to function. Further to this, no homologues of mammalian PRMT5 methylosome components were identified in *Tb*PRMT5 immunoprecipitation experiments (Pasternack et al., 2007).

Under native conditions, *Tb*PRMT5 is found in different protein complexes, with sizes ranging between 150 and 700 kDa (Pasternack et al., 2007). Strikingly, *Tb*PRMT5 interacts with Kinetoplastid-specific proteins, suggesting importance in Kinetoplastid-specific pathways (Pasternack et al., 2007). Further studies are necessary to verify *Tb*PRMT5 substrates and determine consequences of PRMT5-dependent methylation. In addition, sequence data indicates that the currently uncharacterized *Leishmania* PRMT5 is much larger (>1,000 amino acids) than its orthologues in humans and *T. brucei* (637 and 784 amino acids, respectively) due to a much longer N-terminus that contains no known conserved functional domains. Elucidating the 3D structure of at least one trypanosomatid PRMT5 will lend insight into potential functional roles of this N-terminus.

### *Trypanosoma brucei* Protein Arginine Methyltransferase 6

*Tb*PRMT6 is a type I PRMT that displays a narrow substrate specificity. Accordingly, *Tb*PRMT6 knockdown does not visibly alter the cellular arginine methylation profile (Lott et al., 2014). Results suggest that protein targets of *Tb*PRMT6 methylation are important for *T. brucei* cellular replication, given *Tb*PRMT6-KD caused a mild growth defect *in vitro* and led to the appearance of aberrant cells (Fisk et al., 2010).

*Tb*PRMT6 is expressed by both *T. brucei* procyclic and bloodstream forms at equal levels and, despite being primarily cytoplasmic, it interacts with several histones. *Tb*PRMT6 also co-purifies with proteins involved in nucleocytoplasmic transport and RNA processing, indicating it might be important in controlling nucleic acid metabolism and transport (Fisk et al., 2010). Disruption of these processes is known to cause growth defects *in vitro* and *Tb*PRMT6 transcription is reduced in cells exposed to DNA damage (Stortz et al., 2017).

*Tb*PRMT6 contains the four canonical PRMT domains (Figures 2A,C), and holds unique sequence and structural features that appear conserved across the Kinetoplastids. Secondary structure analysis of type I PRMTs indicates that *Tb*PRMT6 contains four insert regions and a truncated C-terminus. The insertions seem to extend or introduce additional α-helices (Wang et al., 2014b), indicating potential relevance to methyltransferase activity and/or regulation, as well as substrate selection. The exact role of these peculiarities is still to be determined.

Importantly, the 3D structure of apo-*Tb*PRMT6 complexed with S-adenosylhomocysteine (SAH) revealed that substrate binding remodels the active site to allow correct positioning of the target arginine residue. These conformational changes involve residues that are conserved in type I PRMTs, including His318 in the THW loop and Glu142 in the Double E loop, suggesting this feature may be conserved among type I enzymes (Wang et al., 2014b).

### *Trypanosoma brucei* Protein Arginine Methyltransferase 7

Kinetoplastids are the only unicellular eukaryotes known to express a PRMT7 homolog. Whereas the mammalian PRMT7 polypeptide contains two copies of the core PRMT fold, which interact with each other to form an intramolecular- or pseudo-dimer (Cura et al., 2014), *Tb*PRMT7 contains only a single active site and is almost half the size of human PRMT7, although much more active (Fisk et al., 2009). *Tb*PRMT7 is a cytoplasmic enzyme expressed by both long-slender bloodstream and procyclic form *Trypanosoma brucei*, and at least in the latter, it forms different macromolecule complexes (Fisk et al., 2009, Fisk et al., 2010). The composition of these complexes is still unknown, but it likely contains RNA, as *Tb*PRMT7 can bind RNA *in vitro* and *in vivo* (Lueong et al., 2016; Kafková et al., 2018).

Curiously, knockdown of *Tb*PRMT7 only minimally affects the MMA profile, which might be due to an increase in the monomethylation activity of other PRMTs (Lott et al., 2014). Simultaneous knockdown of *Tb*PRMT7 and *Tb*PRMT1 reduced both MMA and aDMA levels in the cells, whereas the knockdown of *Tb*PRMT1 alone caused an accumulation of MMA. All evidence suggests that *Tb*PRMT7 generates monomethylated substrates for other PRMTs and that *Tb*PRMT1 activity can compensate for reduction of *Tb*PRMT7 activity (Lott et al., 2014). Accordingly, recombinant *Tb*PRMT7 displays broad substrate specificity *in vitro*, which includes proteins known to be methylated by *Tb*PRMT1 and *Tb*PRMT5, such as RBP16 and *Tb*RGG1 (Fisk et al., 2009). It is therefore possible that *Tb*PRMT7 is also involved in RBP16^Arg93^ constitutive methylation.

The *Tb*PRMT7 3D structure showed the expected four canonical domains of PRMTs (Figures 2A,D). Similar to the other Kinetoplastid PRMTs, homodimerization is facilitated by hydrophobic interactions between a dimerization arm and the SAM-binding domain of the other subunit (Wang et al., 2014a). Extensive mutations on the dimerization arm abolished dimerization, leaving only residual methyltransferase activity (Debler et al., 2016).

Each *Tb*PRMT7 monomer can bind SAM and arginine substrate molecules (Wang et al., 2014a; Debler et al., 2016), though the arginine substrate binding pocket appears to be significantly narrower than those of type I and II PRMTs, consistent with its ‘monomethylation only’ profile. In fact, conserved residues present in the double E and THW loops restrict *Tb*PRMT7 to monomethylation. An E181D mutant was able to catalyze aDMA (Debler et al., 2016), while an E181D/Q329A double mutant generated sDMA (Jain et al., 2016). Simulation studies also indicate that E172 and Q329 are crucial for proper substrate orientation and facilitating the reaction mechanism (Thakur et al., 2019). Furthermore, a F71I mutant was able to form dimethylated products (Jain et al., 2016; Cáceres et al., 2018). These data showed the importance of the double E loop with each of E172 and E181 forming two hydrogen bonds to the guanidino group of the substrate arginine (Wang et al., 2014a) (Figure 2E).

## Protein Arginine Methyltransferase 7 in *Leishmania*

Although arginine methylation in *Leishmania* was observed prior to identification in *T. brucei* (Paolantonacci et al., 1986), the PRMT studies in *Leishmania* are much more limited, primarily focused on *Leishmania major* PRMT7. Notably, different from *Trypanosoma* PRMT3, *Leishmania* PRMT3 displays an intact double E loop (although with mutated THW loop), and might be enzymatically active, a matter for further investigation.

In contrast to *T. brucei* findings, *Lmj*PRMT7-knockout clearly changed the MMA profile in cells, although arginine monomethylation was not abolished (Ferreira et al., 2014). Interestingly, MMA seems less pronounced in the stationary culture phase (containing metacyclic promastigotes), which correlates with the absence of PRMT7 expression. Furthermore, unlike mammalian PRMT7, *Lmj*PRMT7 is a cytoplasmic-specific enzyme. The observation that mitochondrial *Lmj*RBP16 became hypomethylated upon *Lmj*PRMT7-knockout suggests that some substrates are modified before sorting to organelles (Ferreira et al., 2014; Ferreira et al., 2020). Importantly, cytoplasmic *Lmj*RBP16 displays a shorter half-life in the absence of *Lmj*PRMT7, indicating the importance of methylation for its stability (Ferreira et al., 2020).

247 *L. major* proteins were found bearing MMA, of which 40 became hypomethylated and 17 became hypermethylated upon *Lmj*PRMT7 deletion (Ferreira et al., 2020). This suggests that at least 40 proteins are *Lmj*PRMT7 substrates and at least 17 display alternative methylation upon *Lmj*PRMT7 depletion. *Lmj*PRMT7-mediated MMA was enriched in RG/RGG motifs and “Nucleic acid binding” and “RNA binding” were the most enriched functions annotated for the hypomethylated proteins. Fifteen out of the 40 hypomethylated proteins in *Lmj*PRMT7-knockout cells and 75 out of the 247 MMA-carrying proteins were orthologues of *L. mexicana* candidate RBPs (de Pablos et al., 2019; Ferreira et al., 2020).

Although MMA often occur at variable proximity to the RNA-binding domains, it can influence both RBP activity and RNA fate. Absence of *Lmj*PRMT7 reduces *Lmj*Alba3:*δ-amastin* mRNA binding, which caused a ∼4-fold decrease in the half-life of *δ-amastin* transcripts (Ferreira et al., 2020) (Figure 1C). Despite only minor changes in the global transcriptome of *Lmj*PRMT7-KO parasites, this mutation had a clear biological impact as *Lmj*PRMT7-KO cells are more virulent *in vitro* and *in vivo* than wildtype *L. major* (Ferreira et al., 2014; Diniz et al., 2021). Concordantly, the virulent *L. major* LV39 strain expressed lower levels of *Lmj*PRMT7 than the avirulent CC1 strain (Ferreira et al., 2014) and knockout of *Lmj*PRMT7 in the CC1 strain recovered its virulence (Diniz et al., 2021). Curiously, gain of virulence upon *Lmj*PRMT7 deletion was not linked to an increase in parasite burden, but to increased recruitment of neutrophils to the site of infection (Diniz et al., 2021). Further studies will investigate the biological process underlying this altered immune response.

## Concluding Remarks

Our knowledge on trypanosomatid RBPs is still very limited (Clayton, 2013). Although recent high-throughput analyses have expanded the list of actual and potential RBPs in these organisms (Erben et al., 2014; Lueong et al., 2016; de Pablos et al., 2019), many questions remain, particularly concerning how the protein:RNA binding processes are coordinated throughout their lifecycles (de Pablos et al., 2016). In this scenario, the function of PRMTs in regulating post-transcriptional gene expression is of great importance. It has been shown that Kinetoplastid PRMTs interact with proteins from distinct cellular compartments and that their function can impact different biological events, from *in vitro* growth to animal infection. Interference with these processes has been demonstrated to impact parasite fitness. In addition, RBPs enriched in RG/RGG motifs are associated with biomolecular phase separation (Chong et al., 2018). The modification of RG motifs by PRMTs is a mechanism that cells use to regulate formation and dissolution of biomolecular condensates, a phenomenon that has not been widely explored in Kinetoplastids and deserves more attention. Moreover, investigating which other PTMs exist in close proximity to methylated arginine residues and how these functionally interact is of great interest.

The complex functions of Kinetoplastid PRMTs promote their potential as candidate targets for drug or chemical probe development. Although much structural, biochemical, biophysical and inhibition data are still missing for the Kinetoplastid proteins, the structural features known to be specific to Trypanosomatid PRMTs combined with the large inhibitor arsenal targeting human PRMTs may enable the repurposing of drugs and the development of novel anti-parasite strategies. Thus, improving our understanding of the molecular and biological processes that coordinate and are coordinated by PRMT activities in Kinetoplastids is of great relevance for the treatment of diseases caused by these parasites.
